# Editorial: Seizure forecasting tools, biomarkers and devices

**DOI:** 10.3389/fnins.2024.1470640

**Published:** 2024-08-28

**Authors:** Mona Nasseri, Caitlin Grzeskowiak, Benjamin H. Brinkmann, Matthias Dümpelmann

**Affiliations:** ^1^School of Engineering, University of North Florida, Jacksonville, FL, United States; ^2^Neurology Department, Mayo Clinic, Rochester, MN, United States; ^3^Research and Innovation Department, Epilepsy Foundation, Landover, MD, United States; ^4^Epilepsy Center, Department of Neurosurgery, Medical Center, Faculty of Medicine, University of Freiburg, Freiburg, Germany

**Keywords:** seizure forecasting algorithms, seizure forecasting devices, seizure forecasting biomarkers, data collection, epilepsy

Recent advancements in data collection through seizure diaries, invasive and non-invasive devices, and implementing computational algorithms for identifying seizure patterns and likelihood such as statistical and machine learning algorithms have made reliable seizure forecasting increasingly feasible (Meisel et al., 2020; Brinkmann et al., 2021; Nasseri et al., 2021; Stirling et al., 2021). This holds great promise for improved seizure management. However, several significant challenges remain such as high false alarm rates that undermine the reliability of current forecasting algorithms (Karoly et al., 2020), and the variability in seizure patterns among individuals, which makes it difficult to create a generalized model. Additionally, the quality of recorded physiological data is crucial (Nasseri et al., 2020; Böttcher et al., 2022); while non-invasive devices can provide valuable data, they are not always acceptable to all patients. Integrating various types of data, such as physiological signals, sleep patterns, and mood information, into a single forecasting model requires substantial data processing power and sophisticated algorithms. Moreover, implementing seizure forecasting in real-life conditions necessitates evaluating the acceptability of algorithm performance metrics among patients and clinicians (Grzeskowiak and Dumanis, 2021). These challenges underscore the need for ongoing research and innovation in the field of seizure forecasting.

One of the primary challenges with current seizure forecasting algorithms is their high false alarm rate (FAR), which limits clinical applicability and acceptability to patients (Alotaiby et al., 2014; Beniczky et al., 2021). High FAR is a critical barrier to the widespread clinical adoption and utility of seizure forecasting technologies necessitating development of more robust and accurate prediction models. In the study Segal et al. researchers applied a risk-controlling prediction (RCP) calibration method called Learn then Test (LTT) to address this issue. The calibration algorithm was first validated with synthetic data and then tested on scalp EEG recordings. A convolutional neural network was used to assess seizure risk by defining the preictal state as the period from 60 min to 30 s before seizure onset. By implementing LTT as a post-processing step on the test dataset, an average reduction of 92% in the FAR was achieved. Although this calibration method improved model performance for some recordings, the results were still not sufficient for clinical relevance.

On the other hand, to develop reliable forecasting algorithms, identifying biomarkers and features that capture the non-linear and non-stationary nature of physiological signals is another promising area of investigation (Brinkmann et al., 2016; Meisel et al., 2020; Nasseri et al., 2021). A 2014 paper (Wang and Lyu, 2014) derived comprehensive feature vectors from EEG signals for efficient prediction and examined the relevance of primary amplitude and frequency components to a patient's seizure occurrence. The study by Chen et al., highlights the significance of time-frequency features extracted from cEEG in forecasting seizures. Time-frequency and power spectrum analysis were applied to investigate periodic discharges (PDs) patterns and their relation to seizure prediction. The study demonstrated that high spectral power predicted a high risk of seizures, while low spectral power was associated with a lower risk. However, a prospective study that includes a larger cohort of patients is needed to confirm these findings.

During the first international seizure prediction workshop, the seizure prediction community established standards for datasets to be used in the development and evaluation of seizure prediction algorithms (Lehnertz and Litt, 2005). As a result, several EEG datasets adhering to these guidelines were created and are recommended for research groups working in the area of seizure prediction (Wagenaar et al., 2015). Further datasets were made available by the community (Brinkmann et al., 2016; Kuhlmann et al., 2018). In the current Research Topic, Andrade et al. compared the performance of a patient-specific seizure prediction algorithm across four open access databases in a standardized way. The study distinguishes between sample-based approaches disregarding the temporal aspect of seizures, and alarm-based approaches, which aim to simulate real-life conditions. In this more realistic scenario results were less promising and shows the importance of rigorous testing conditions. However, the existing databases also have deficits, as they contain EEG recordings of only a few seizures for many patients. Datasets with ultra-long term recordings with subcutaneous (Pal Attia et al., 2023; Viana et al., 2023) or intracranial EEG (Kuhlmann et al., 2018) offer the possibility to overcome some of these limitations, although these continuous ultra-long-term datasets remain rare due to the challenges in acquiring such data. Efforts are underway to overcome these barriers (Khambhati et al., 2024).

A lower burden for patients is achieved by using wearable devices or intelligent (sensor-integrated) clothing during daily routines. Gagliano et al. used a smart shirt with a single-lead ECG, two respiratory bands and a detachable telemetry device with a three-axis accelerometer to investigate the relation between sleep efficiency and the occurrence of epileptogenic seizures. Relationships between vigilance states, sleep stages and epilepsy are known for many years and circadian profiles of seizure occurrence are not only results of recent research (Khan et al., 2018) but are still not completely understood and remain an active Research Topic, particularly in relation to ictal and interictal EEG activity (Peter-Derex et al., 2020). In this study, four sleep metrics were derived from the sensor data of the smart shirt: total sleep duration, sleep latency, time of waking after sleep onset, and sleep efficiency. It could be shown, that sleep quality, especially sleep efficiency is lower in nights before a seizure. These results complement those obtained using a wristband wearable (Stirling et al., 2023), showing that sleep onset and offset times were significantly associated with heightened seizure risk the following day for more participants than changes in sleep duration the night before. Overall these studies emphasize the richness of information, which can be derived from mobile recording devices contributing to seizure forecasting in real life settings.

Seizure forecasting holds the promise of significantly reducing the burden of epilepsy and enhancing the autonomy and quality of life for those affected. To fully realize this potential, further improvements in accuracy, reduction of patient burden, and integration with targeted interventions are essential.
